# Broad spectrum developmental role of Brachypodium AUX1

**DOI:** 10.1111/nph.15332

**Published:** 2018-06-27

**Authors:** Alja van der Schuren, Catalin Voiniciuc, Jennifer Bragg, Karin Ljung, John Vogel, Markus Pauly, Christian S. Hardtke

**Affiliations:** ^1^ Department of Plant Molecular Biology University of Lausanne Biophore Building CH‐1015 Lausanne Switzerland; ^2^ Institute for Plant Cell Biology and Biotechnology Heinrich‐Heine University D‐40225 Duesseldorf Germany; ^3^ DOE Joint Genome Institute 2800 Mitchell Dr. Walnut Creek CA 94598 USA; ^4^ Umeå Plant Science Center Department of Forest Genetics and Plant Physiology Swedish University of Agricultural Sciences SE‐901 83 Umeå Sweden

**Keywords:** AUX1, auxin, Brachypodium, monocotyledon, seminal root

## Abstract

- Targeted cellular auxin distribution is required for morphogenesis and adaptive responses of plant organs. In *Arabidopsis thaliana* (Arabidopsis), this involves the prototypical auxin influx facilitator AUX1 and its LIKE‐AUX1 (LAX) homologs, which act partially redundantly in various developmental processes. Interestingly, *AUX1* and its homologs are not strictly essential for the Arabidopsis life cycle. Indeed, *aux1 lax1 lax2 lax3* quadruple knock‐outs are mostly viable and fertile, and strong phenotypes are only observed at low penetrance.
- Here we investigated the *Brachypodium distachyon* (Brachypodium) *AUX1* homolog *BdAUX1* by genetic, cell biological and physiological analyses.
- We report that *BdAUX1* is essential for Brachypodium development. *Bdaux1* loss‐of‐function mutants are dwarfs with aberrant flower development, and consequently infertile. Moreover, they display a counter‐intuitive root phenotype. Although *Bdaux1* roots are agravitropic as expected, in contrast to Arabidopsis *aux1* mutants they are dramatically longer than wild type roots because of exaggerated cell elongation. Interestingly, this correlates with higher free auxin content in *Bdaux1* roots. Consistently, their cell wall characteristics and transcriptome signature largely phenocopy other Brachypodium mutants with increased root auxin content.
- Our results imply fundamentally different wiring of auxin transport in Brachypodium roots and reveal an essential role of *BdAUX1* in a broad spectrum of developmental processes, suggesting a central role for AUX1 in pooideae.

## Introduction

Modulation of auxin activity through differential auxin distribution plays a central role in developmental and adaptive growth processes (Benjamins & Scheres, 2008; Zazimalova *et al*., 2010). It is largely achieved through plasma membrane‐integral auxin efflux carriers, the PIN‐FORMED (PIN) proteins, whose polar cellular localization can lead to asymmetric auxin secretion. Coordination of PIN polarity across cell files thus can promote targeted, so‐called polar auxin transport at the tissue and organ level (Benjamins & Scheres, 2008; Zazimalova *et al*., 2010). In contrast to the carrier requirement for auxin efflux, cellular auxin influx can occur through diffusion, because in the acidic environment of the apoplast auxin is mostly protonated and thus lipophilic enough to cross the plasma membrane (Zazimalova *et al*., 2010). Nevertheless, dedicated auxin influx facilitators, AUX1 and the LIKE AUX1 (LAX) proteins that accelerate auxin uptake have been identified (Maher & Martindale, 1980; Bennett *et al*., 1996; Marchant *et al*., 2002; Yang *et al*., 2006; Peret *et al*., 2012). Their differential expression, as well as often polar localization, can modulate polar auxin transport to reinforce or attenuate local auxin accumulations. *Arabidopsis thaliana* (Arabidopsis) mutants in the prototypical auxin influx facilitator AUX1 have been identified because of their root agravitropism (Maher & Martindale, 1980), which can be rescued by addition of the lipophilic auxin analog 1‐naphthylacetic acid (1‐NAA) (Swarup *et al*., 2001). Mutants in the three *AUX1* homologs, *LAX1‐3*, display either no, or less conspicuous phenotypes (Ugartechea‐Chirino *et al*., 2010; Vandenbussche *et al*., 2010; Peret *et al*., 2012). However, corresponding multiple mutants reveal (partially) redundant roles of AUX1 and LAX1‐3, for instance in phyllotaxis (Bainbridge *et al*., 2008) and embryogenesis (Robert *et al*., 2015), although mutant phenotypes are not always fully penetrant. Moreover, AUX1 and LAX1‐3 proteins are not fully interchangeable in every cellular context (Peret *et al*., 2012).

**Figure 1:**
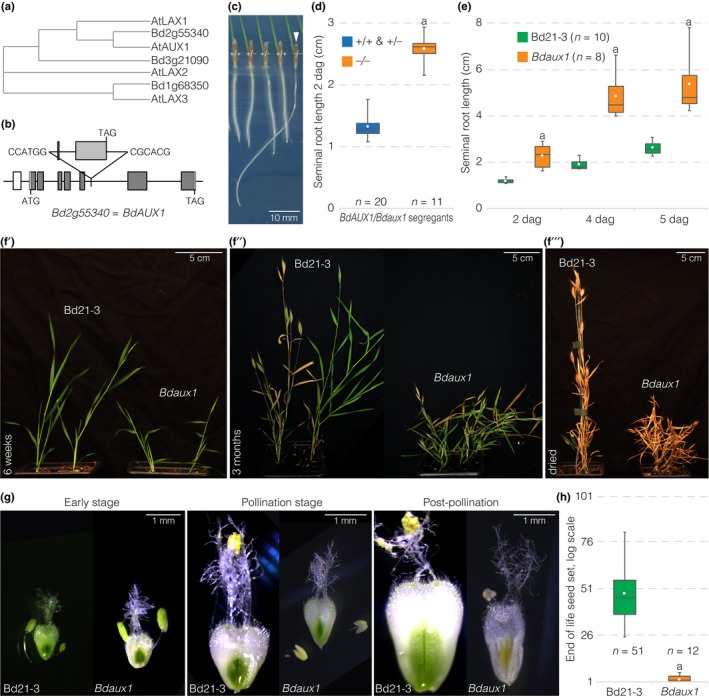
Root and shoot phenotypes of the *Bdaux1* mutant. (a) Sequence similarity (Clustal alignment, neighbor joining, with distance correction) of Arabidopsis and Brachypodium AUX1 homologs. (b) Schematic presentation of the T‐DNA insertion line for *BdAUX1*. (c) Representative seedlings (2‐d‐old) segregating in the progeny of a heterozygous *BdAUX1/Bdaux1* (±) mother plant, genotypes are indicated. (d, e) Seminal root length of indicated genotypes (dag, days after germination). (f) Shoot development of *Bdaux1* plants in comparison to its Bd21‐3 wild type background at different stages of the life cycle. (g) Different stages of flower development in *Bdaux1* plants as compared to Bd21‐3. (h) End of life seed set in indicated genotypes. Box plots display second and third quartiles, maximum, minimum and mean (white dot). Statistically significant differences are indicated (Student's *t*‐test; a, *P *<* *0.001).

Compared to the well characterized roles of AUX1/LAX1‐3 in Arabidopsis, little is known about the developmental role of auxin influx facilitators in monocotyledons (Balzan *et al*., 2014). Yet, AUX1 homologs can be readily identified, since they are highly conserved. For example, in rice (*Oryza sativa*) and the more distantly related panicoid grasses maize (*Zea Mays* L.) and *Setaria viridis* (Setaria), five AUX1 homologs have been identified (Zhao *et al*., 2012; Huang *et al*., 2017). In maize, the closest *AtAUX1* homolog has 73% sequence identity (Hochholdinger *et al*., 2000). Functional studies of mutants in *AUX1* homologs in maize and Setaria demonstrated involvement of those genes in inflorescence development and root gravitropism (Huang *et al*., 2017). Also, the *OsAUX1* gene has subsequently been implicated in lateral root formation and shoot elongation (Zhao *et al*., 2015), as well as seminal root elongation and root hair elongation (Yu *et al*., 2015). Although rice, maize and Setaria can be considered model systems for the grasses, it remains unclear whether findings from these species can be directly transferred to other groups within the poaceae. One such group is the pooideae, which comprise the major cereal crops wheat, rye and barley. The monocotyledon *Brachypodium distachyon* (Brachypodium) is a model species for these temperate cereals (Brkljacic *et al*., 2011; Girin *et al*., 2014). AUX1 homologs can be readily identified in the Brachypodium genome. However, unlike rice, maize or Setaria with five homologs, Brachypodium only possesses three AUX1 homologs, which display almost sequence identity with their Arabidopsis counterparts (Supporting Information Fig. S1). Nevertheless, slightly divergent N‐ and C‐termini and the gene sequences allow the assignment of clear one‐to‐one homologies in sequence similarity analyses (Fig. 1a). Here we investigated the developmental role of the closest AUX1 homolog of Brachypodium, the *Brachypodium distachyon AUX1 (BdAUX1)* gene. We report that *BdAUX1* loss‐of‐function results in counter‐intuitive root phenotypes and reveals its essential role in a broad spectrum of developmental processes, suggesting a more central and diversified role for AUX1 in pooideae.

## Materials and Methods

### Plant materials, genotyping and growth conditions

The *Bdtar2l* ^*hypo*^ mutant has been described before (Pacheco‐Villalobos *et al*., 2013). The *Bdaux1* mutant line JJ5658 was obtained from a Brachypodium T‐DNA insertion library (Bragg *et al*., 2012). RT‐PCR was performed to verify that the T‐DNA insertion indeed leads to a truncated *BdAUX1* mRNA. To this end, the following oligonucleotides were used: F1 5′‐ATG GTG CCG CGC GAG CAT G‐3′, located at the start‐codon; R1 5′‐GCA TGA TCT CCA CTG TGA CG‐3′, at the border of the T‐DNA insertion; R2 5′‐GGT GAA GCT GAC GAG TAG CG‐3′, located 285 bp before the STOP‐codon; and R3 5′‐ GAT CCG GTA GTT GTG GAA GG‐3′, located 160 bp before the T‐DNA insertion (see Fig. S2A). *Bdaux1* ^*CRISPR*^ mutants were obtained directly as homozygotes from transformations (see below, ‘Transformation’) and could not be amplified due to their sterility. *Bdtar2l* ^*hypo*^ *Bdaux1* double mutants were obtained by crossing. For tissue culture, seeds were sterilized as described (Bragg *et al*., 2012) and stratified for 3 d at 4°C before transfer to plates with half‐strength Murashige‐Skoog (MS) media (2.45 g l^−1^ MS salts with vitamins, 0.3% sucrose, 1% agar, pH 5.7) placed vertically at a slight angle to prevent roots from growing into the media or the air. Unless indicated otherwise, analyses were performed on 2‐d‐old seedlings raised as previously described (continuous light of 100–120 μE intensity, 22°C, PhilipsF17T8/TL741 fluorescent light bulbs) (Pacheco‐Villalobos *et al*., 2013). Roots that had grown into the media or the air were excluded from analysis. For gravitropism assays, seeds were grown for 1 d on vertically oriented plates, which were then rotated 90° and seedlings were left to grow for another 2 d. Root length was measured using Fiji software (https://imagej.net/Fiji?Downloads). For auxin analysis, cell wall analysis and RNAseq, 1 cm seminal root segments harvested 2–3 mm above the root tip were used (Pacheco‐Villalobos *et al*., 2016). Genotyping of *Bdtar2l* ^*hypo*^ was performed as described (Pacheco‐Villalobos *et al*., 2013). For *Bdaux1* genotyping, the wild type allele was monitored with primers 5′‐GTG AAC TTT CCA CAC TGA GC‐3′ and 5′‐TCA CAA GAG CTG GGC AAT GG‐3′, and the T‐DNA insertion with 5′‐GTG AAC TTT CCA CAC TGA GC‐3′ and 5′‐CAG GAA TTC ATG CCG ACA GC‐3′. Double mutants were genotyped with the same methodology for both T‐DNA insertions.

### Plasmid construction

To create a vector with kanamycin resistance, the *nptII* sequence was amplified with primers 5′‐CCA CTC GAG GAT CTC CAC TCT AGT CGA G‐3′ and 5′‐TGT CTC GAG TTG AAC GAT CGG GGA TCC‐3′. The fragment was digested with *Xho*I and cloned into *Xho*I‐digested pCAMBIA1305.1 to replace the hygromycin‐resistance gene to give pCAMBIA1305.1‐nptII. Next, *BdAUX1::BdAUX1* was amplified in three pieces from genomic DNA with primers 5′‐CAT GAT TAC GAA TTC GAG CTC GTC ACT TAA TCT CGT C‐3′ and 5′‐CGA ATT TCC TCT CTG TCT CC‐3′ for piece 1, 5′‐GGA GAC AGA GAG GAA ATT CG‐3′ and 5′‐CAA TGC ACC TCA TCG TTC CA‐3′ for piece 2, and 5′‐CAA TGC ACC TCA TCG TTC CA‐3′ and 5′‐GGA AAT TCG AGC TGG TCA CCT AGC AAG CAT TAC TGG GTT‐3′ for piece 3. The fragments were combined into *Sac*I–*Sal*I‐digested pCAMBIA1305.1‐nptII using Gibson ligation. *BdAUX1::NLS3xVENUS* was created by insertion of amplified *NLS3xVENUS* into *Hin*dIII–*Pml* I‐digested pCAMBIA1305.1‐nptII. The *BdAUX1* promoter was then amplified with primers 5′‐CTA GAG CTC TGG ACG TGG TTT TGT CCT AG‐3′ and 5′‐ACG CGT CGA CAT CTC TTC AAC GCG CTG TC‐3′, and inserted in front of *NLS3xVENUS* using *Sac*I and *Sal*I digestion. For BdAUX1 localization, a GFP fusion tag was added to the protein. To this end, *BdAUX1* promoter was amplified with primers 5′‐GCG ACT GTG CCA ACA CCC‐3′ and 5′‐GCC CTT GCT CAC CAT CTC TTC AAC GCG CTG TCC TC‐3′, the transcript region was amplified with primers 5′‐GTC GAC TCT AGA GGA TCC ATG GTG CCG CGC GAG CAT‐3′ and 5′‐TTT TTC CTC GGG TTA GTT AAT TAA TTC‐3′, and GFP was amplified from pVec8GFP with primers 5′‐ATG GTG AGC AAG GGC GAG G‐3′ and 5′‐ATC CTC TAG AGT CGA CCT TGT ACA GCT CGT CCA TGC‐3′. The three fragments were then combined into *Xma*I–*Pac*I‐digested pCAMBIA1305.1‐nptII in a Gibson reaction. The *BdAUX1* CRISPR/Cas9 cassette was created by amplifying the *Zea mays UBIQUITIN (UBQ)* promoter (Bragg *et al*., 2012) using primers 5′‐GAG CTC CAG CTT GCA TGC CTG CAG TG‐3′ and 5′‐GAG CTC TCT AGA GTC GAC CTG CAG AA‐3′ and ligation of the fragment into *Sac*I‐digested pCAMBIA1305.1. A *Brachypodium*‐optimized Cas9 with FLAG‐tag and nuclear localization signal (Methods S1), followed by a multiple cloning site, was synthesized and cloned behind the *UBQ* promoter after *Kpn*I and *Bst*eII digestion, to create vector p5Cas. Next, a 770 bp cassette containing a *Brachypodium U6* promoter, *Bsa*I restriction sites, tracrRNA, a rice *U6* promoter, *Btg*ZI restriction sites and tracrRNA was synthesized (see Methods S1) and cloned into *Bam*HI–*Eco*RI‐digested pDONR221. This allowed two sgRNA sequences to be added, using *Bsa*I and *Btg*ZI restriction sites, respectively. The *Bdaux1* knock‐out cassette was then assembled by annealing, phosphorylating and ligating the following primer pairs into the *Bsa*I–*Btg*ZI‐digested pDONR vector: 5′‐TCT CGT CAC CAG CTT CCT CTG GCA‐3′ and 5′‐AAA CTG CCA GAG GAA GCT GGT GAC‐3′ for sgRNA1, and 5′‐GTG TGA TCC GGT AGT TGT GGA AGG‐3′ and 5′‐AAA CCC TTC CAC AAC TAC CGG ATC‐3′ for sgRNA2. The sgRNA cassette was then isolated and ligated into p5Cas via *Bam*HI–*Hin*dIII restriction digest. Target specificity of the sgRNA was checked bioinformatically (http://bioinfogp.cnb.csic.es/tools/breakingcas/?gset=8x2_GENOMES_EnsemblGenomes_39).

### Transformation

For Brachypodium transformations (Pacheco‐Villalobos *et al*., 2013) the *Agrobacterium tumefaciens* strain GV3101 pMP90 was used. *BdAUX1::NLS‐3XVENUS*,* BdAUX1::BdAUX1* and *BdAUX1::GFP‐BdAUX1* transformants in Bd21‐3 and *Bdaux1* were selected on media with 400 μg ml^−1^ paramomycin and 600 μg ml^−1^ CuSO_4_. Regeneration media contained 50 μg ml^−1^ paramomycin and 600 μg ml^−1^ CuSO_4_. Transformants for the CRISPR/Cas9 *BdAUX1* knock out construct were selected on hygromycin as described (Pacheco‐Villalobos *et al*., 2013), with the addition of 600 μg ml^−1^ copper sulfate (CuSO_4_) to the regeneration media.

### Metabolic analyses, qPCR and RNAseq

For auxin measurements, three independent batches of two replicates each, containing 20 pooled 1‐cm root segments per genotype were analyzed as described (Pacheco‐Villalobos *et al*., 2013, 2016). For cell wall polysaccharide quantifications, three independent pools of 100 to 120 segments per genotype were collected and freeze‐dried overnight. The monosaccharide composition and glycosidic linkages of the wall material was analyzed as described (Pacheco‐Villalobos *et al*., 2016). qPCR on Brachypodium *AUX1* homologs was performed as described normalizing against *UBIQUITIN CONJUGATING ENZYME 18 (BdUBC18)* (Pacheco‐Villalobos *et al*., 2013). The following specific primers were used: 5′‐CCA TGT CAT CCA GTG GTT CG‐3′ and 5′‐GAT GAG CTG GAT GAC GGA GC‐3′ for Bradi1g68350; 5′‐CGT CAT CCA GTG GTT TGA GG‐3′ and 5′‐CAG CCG ATG AGC TGG ATC AC‐3′ for Bradi3g21090. For RNAseq, two independent pools of segments were collected from 12 roots per genotype. RNAseq was performed as described (Pacheco‐Villalobos *et al*., 2016). The raw data have been deposited in the NCBI Sequence Read Archive (https://www.ncbi.nlm.nih.gov/sra/) under accession SRP137652.

### Microscopy

For microscopic imaging, seminal roots of 2‐d‐old seedlings were fixed 1 wk in 4% (w/v) paraformaldehyde in 1× phosphate‐buffered saline (PBS) solution (pH 6.9). Roots were then washed two times in 1× PBS before transfer into ClearSee solution for at least one month, which was necessary to quench the challenging autofluorescence of Brachypodium roots. ClearSee solution was changed weekly. Then, 2–3 d before imaging, roots were stained with 0.2% Calcofluor White (in ClearSee) solution for 1–2 h with gentle shaking, next washed in ClearSee solution until imaging. Root hairs were imaged in differential interference contrast using a Leica DM5000 microscope. For meristem analyses, stained roots were mounted in ClearSee solution and imaged with Zeiss 880 or LSM710 inverted confocal microscopes using ×40 oil objectives. For Calcofluor imaging, roots were excited with a 405 nm laser and emission signal was captured over 410–509 nm. GFP was imaged with sequential scans using the 518 nm Argon laser and a 493–523 nm emission spectrum to reduce background. NLS‐3×VENUS was imaged as a sequential scan and excited with a 488 nm laser, emission was recorded at 519–572 nm to reduce background. Cell length measurements were performed with Fiji software.

### Microtome sectioning and analysis

Seminal roots of 2‐d‐old seedlings were fixed overnight at 4°C in 1% glutaraldehyde, 4% formaldehyde and 50 mM sodium phosphate buffer (pH 7.2). Roots were dehydrated for at least 1 h each in 15%, 30%, 50%, 70%, 85% and 100% ethanol (EtOH). Samples were pre‐incubated and embedded in TechnoVit 7100 solution as described (Pacheco‐Villalobos *et al*., 2013). 0.3‐μm sections were obtained on a Leica RM2255 microtome. Sections were stained with 0.1% toluidine blue before visualization with a Leica DM5000 microscope. Cell numbers were counted in one representative image per root using the cell counter plugin of ImageJ software. (https://imagej.nih.gov/ij/plugins/cell-counter.html)

## Results and Discussion

To investigate the role of auxin influx facilitators in Brachypodium, we obtained a T‐DNA insertion line in Bradi2g55340 (*BdAUX1* hereafter), the closest homolog of Arabidopsis *AUX1* (*AtAUX1*) in Brachypodium. In this *Bdaux1* mutant allele, *BdAUX1* is disrupted by an insertion in the 6^th^ intron, which leads to a truncated mRNA (Figs 1b, S2). Plants that were homozygous for this insertion displayed agravitropic roots (Fig. 1c), similar to *Ataux1* loss‐of‐function mutants (Maher & Martindale, 1980; Bennett *et al*., 1996). Thus, the T‐DNA insertion apparently results in *BdAUX1* loss of function. However, unlike *Ataux1* mutants, *Bdaux1* mutant roots were considerably longer than those of their wild type siblings or the corresponding Bd21‐3 wild type background line (Figs 1c–e, S3A). Quantitative RT‐PCR (qPCR) suggested that this phenotype was not due to possible (over)compensatory up‐regulation of the two other *AUX1* homologs in Brachypodium (Fig. S2B). *Bdaux1* plants also displayed a dwarf shoot phenotype with aberrant flower development (Fig. 1f,g). *Bdaux1* mutants were thus sterile (Fig. 1h) and could not be maintained as homozygotes in practice. Both the root and shoot phenotypes could be complemented by introduction of transgenes that expressed either BdAUX1 or GFP‐BdAUX1 fusion protein under control of the native *BdAUX1* promoter *(BdAUX1::BdAUX1* and *BdAUX1::GFP‐BdAUX1)* into the *Bdaux1* background (Fig. S3B,C). Moreover, the mutant phenotypes were also observed in *Bdaux1* homozygous knock out plants that were generated by the CRISPR/Cas9 technique (*Bdaux1* ^*CRISPR*^). This included the severe shoot phenotype and infertility (Fig. S3C,D), which also precluded recovery of the lines. Therefore, *Bdaux1* loss‐of‐function was causative for the observed mutant phenotype.

**Figure 2:**
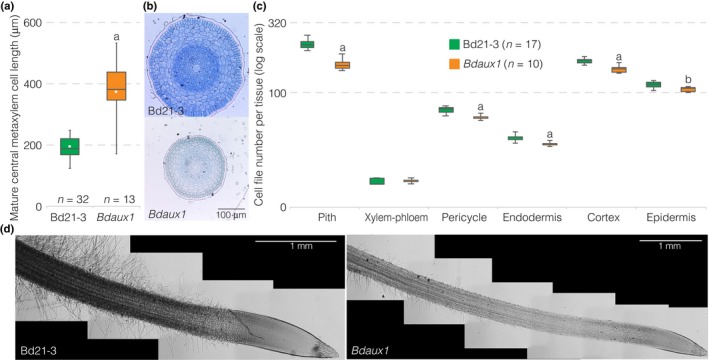
Cellular root phenotypes of the Brachypodium *Bdaux1* mutant. (a) Mature central metaxylem cell length in indicated genotypes. (b) Histological cross‐sections (toluidine blue‐stained) through 2‐d‐old roots of indicated genotypes, taken from the mature part of the root, above the elongation zone. (c) Quantification of cell files in different mature tissue layers of indicated genotypes (2‐d‐old roots). (d) Illustration of root hair development in indicated genotypes (light microscopy, differential interference contrast; composite images). Box plots display second and third quartiles, maximum, minimum and mean (white dot). Statistically significant differences are indicated (Student's *t*‐test: a, *P *<* *0.001; b, *P *<* *0.03).

**Figure 3:**
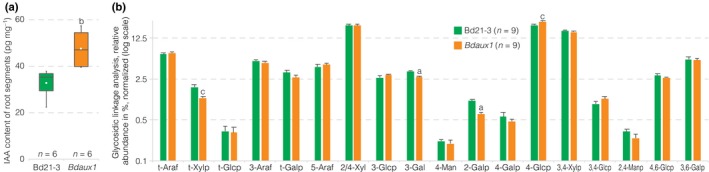
Metabolic Brachypodium *Bdaux1* phenotypes. (a) Free auxin (indole‐3‐acetic acid, IAA) content of 1‐cm root elongation zone segments (2‐d‐old roots) of indicated genotypes. (b) Glycosidic linkage analysis of wall material of 1‐cm root elongation zone segments (2‐d‐old roots) of indicated genotypes (error bars, + standard error (SE)). Box plots display second and third quartiles, maximum, minimum and mean (white dot). Statistically significant differences are indicated (Student's *t*‐test: a, *P *<* *0.001; c, *P *<* *0.05).

A more detailed characterization of the mutants revealed that their increased root elongation could be attributed to increased mature cell length (Fig. 2a). Moreover, *Bdaux1* roots were markedly thinner than wild type roots (Fig. 2b). Although the number of cell files was significantly reduced in every tissue except xylem and phloem (Fig. 2c), this alone could not entirely account for the overall reduction in root thickness. Rather, cells generally appeared slightly smaller in radial sections (Fig. 2b), and at the same time, root hairs were markedly shorter, reduced in number and appeared later than in wild type (Fig. 2d). Therefore, the *Bdaux1* root elongation phenotype was apparently caused by overall higher cellular anisotropy. Interestingly, it thus resembles the roots of hypomorphic mutants in the Brachypodium *TAR2‐LIKE* (*TAR2L*) gene (Pacheco‐Villalobos *et al*., 2013). *Bdtar2l* ^*hypo*^ mutants are partially impaired in a rate‐limiting step of auxin biosynthesis, which results in higher cellular auxin levels in the root because of the particular regulatory wiring in Brachypodium (Pacheco‐Villalobos *et al*., 2013, 2016). To further explore the similarity between *Bdaux1* and *Bdtar2l* ^*hypo*^ mutant roots, we also determined cellular auxin levels in *Bdaux1* root tips. Indeed, we again observed increased auxin levels (Fig. 3a). This result was surprising, given the Arabidopsis precedent that AUX1 is needed for efficient shoot to root mobilization of auxin, and *Ataux1* mutants therefore have reduced, rather than increased, auxin levels in the root (Marchant *et al*., 2002). In *Bdtar2l* ^*hypo*^ plants, the root phenotype was also associated with slight alterations in cell wall composition, notably a reduction in 1,3‐galactosyl and 1,2‐galactosyl residues, suggesting an altered arabinogalactan structure, and an increase in 1,4‐glucosyl residues (Pacheco‐Villalobos *et al*., 2016). Similar changes were observed in *Bdaux1* root tips (Figs 3b, S3E), again confirming similarity with *Bdtar2l* ^*hypo*^ plants. Finally, a survey of the *Bdaux1* transcriptome in elongating root tip segments revealed a number of differentially expressed genes, mostly in cell wall modifiers (Table S1), which were *c*. 10‐fold over‐represented (*P *=* *2.33E‐5). Again, this observation matches what has been described for *Bdtar2l* ^*hypo*^root segments (Pacheco‐Villalobos *et al*., 2016), although the scope of transcriptional changes was less dramatic in *Bdaux1*. A notable commonality was the strong upregulation of expansins, which are thought to be primary targets of auxin‐induced cell elongation (Cosgrove, 2005). Confirming the qPCR analysis, no differential expression of the two other *AUX1* homologs was observed in the *Bdaux1* transcriptome (Table S2). In summary, in many ways *Bdaux1* roots phenocopy *Bdtar2l* ^*hypo*^ roots.

Similarities with *Bdtar2l* mutants could also be observed in the shoot. In mutants of the hypomorphic *Bdtar2l* ^*hypo*^ allele, the root phenotype is accompanied by a slight reduction in leaf blade length and width (Pacheco‐Villalobos *et al*., 2013). However, in mutants of the null allele *Bdtar2l* ^*qnull*^, the root phenotype is weaker and transient, while the shoot displays a dwarf phenotype that is accompanied by severely reduced fertility (Pacheco‐Villalobos *et al*., 2013). Thus, the shoot phenotype of *Bdtar2l* ^*qnull*^ plants is similar to *Bdaux1* plants. The strongly reduced fertility of *Bdaux1* appeared to be due to delayed development of anthers as compared to gynoecia as well as poor pollen viability (Fig. 1g). Nevertheless, because plants heterozygous for *Bdaux1* were similar to wild type, we could create double mutants with the *Bdtar2l* ^*hypo*^ allele. Overall, the phenotype of these *Bdaux1 Bdtar2l* ^*hypo*^ double mutants appeared to be additive as compared to their segregating single mutants and wild type siblings (with the caveat that background loci might modulate the phenotypes to some degree because the two single mutants had different wild type parents). The dwarfism of *Bdaux1* plants was more exaggerated in *Bdaux1 Bdtar2l* ^*hypo*^ double mutants (Fig. S3B,F), and the double mutant roots were thinner than in either single mutant and longer than in *Bdtar2l* ^*hypo*^ alone (Fig. S3G). This could be attributed to an even higher mature cell length, and an additional reduction in cell files (Fig. S2H). However, unlike the single mutants, the double mutants displayed a reduced root meristem size that was accompanied by slight changes in root meristem organization, such as an apparently smaller quiescent center (Fig. S3I,J). Overall, the data suggest parallel impacts of *BdAUX1* and *BdTAR2L* mutation that reinforce each other. This is also consistent with the absence of significant expression changes in rate‐limiting auxin biosynthesis genes in *Bdaux1* (Table S2).

**Figure 4:**
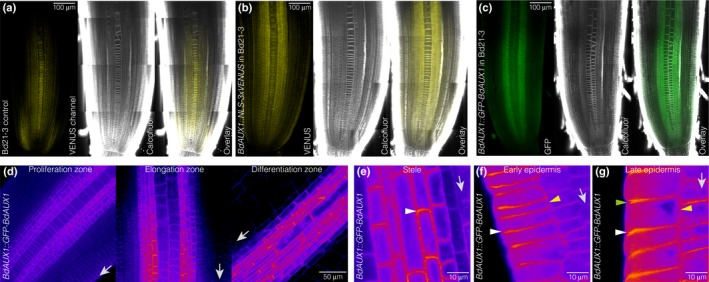
Brachypodium *BdAUX1* expression. (a) Confocal microscopy of a 2‐d‐old Bd21‐3 root meristem after ClearSee and calcofluor staining (white), illustrating background fluorescence (yellow) in the VENUS channel. Please note that autofluorescence of Brachypodium roots cannot be fully eliminated (see the Materials and Methods section). (b) Expression pattern of a *BdAUX1* transcriptional reporter (nuclear‐localized VENUS fluorescence, yellow). (c) Expression pattern of a GFP‐BdAUX1 translational reporter fusion protein (plasma membrane‐localized green fluorescence). (d) Expression level and cellular localization of GFP‐BdAUX1 fusion protein (magenta fluorescence) in different parts of a 2‐d‐old root meristem (arrows point towards root tip). (e) Cellular localization of GFP‐BdAUX1 fusion protein (magenta fluorescence) in the stele, showing shootward polar accumulation of BdAUX1 (arrowhead) (arrow points towards root tip). (f) Cellular localization of GFP‐BdAUX1 fusion protein (magenta fluorescence) in the early epidermis, showing rootward polar accumulation of BdAUX1 (white arrowhead) and absence from inward facing side (yellow arrowhead) (arrow points towards root tip). (g) Cellular localization of GFP‐BdAUX1 fusion protein (magenta fluorescence) in the late epidermis, showing both rootward (white arrowhead) and shootward polar accumulation (green arrowhead), as well as inward facing localization (yellow arrowhead) of BdAUX1 (arrow points towards root tip). (a–c) are composite images.

**Figure 5:**
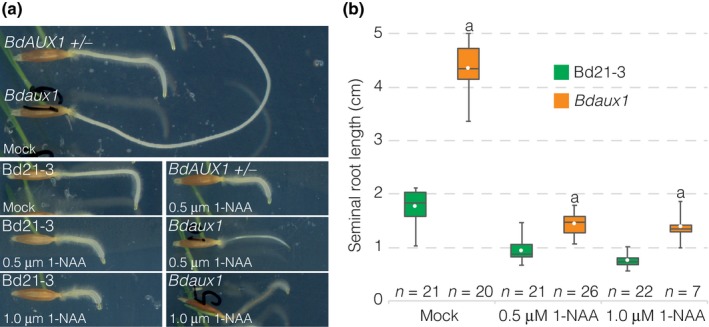
Rescue of Brachypodium *Bdaux1* agravitropism. (a) Response of indicated genotypes to a 90° change in the gravity vector (3‐d‐old roots, plates were turned when they were 1‐d‐old), in the absence or presence of 1‐NAA. (b) Root length of indicated genotypes in the absence or presence of 1‐NAA. Box plots display second and third quartiles, maximum, minimum and mean (white dot). Statistically significant differences are indicated (Student's *t*‐test: a, *P *<* *0.001; b, *P *<* *0.01; c, *P *<* *0.05).

The *BdAUX1::GFP‐BdAUX1* plants, as well as *BdAUX1::NLS‐3XVENUS* plants, allowed us to assess the expression pattern of *BdAUX1* in the root. *AtAUX1* is expressed specifically in the Arabidopsis root protophloem, epidermis and root cap‐columella (Marchant *et al*., 2002). *BdAUX1* transcriptional and translational reporters displayed similar expression patterns, with the exception of expression in the root cap. Moreover, unlike *AtAUX1*,* BdAUX1* was also expressed throughout the stele and in the outer cortex layers (Fig. 4a–c). Thus, the expression pattern of *BdAUX1* encompasses the combined domains of *AtAUX1*,* AtLAX2* and *AtLAX3* (Peret *et al*., 2012) with the exception of the root cap, and therefore, possibly, their combined functions in these tissues. Consistent with its homology to AtAUX1, GFP‐BdAUX1 protein was localized at the plasma membrane, in a typically polar fashion (Fig. 4d,e). In the stele, the orientation was generally shootward (Fig. 4e), while in the outer cell layers, BdAUX1 polar localization appeared mostly rootward (Fig. 4f). However, in the later epidermis, BdAUX1 was detected on both the apical and basal sides of the cell, as well as facing inside (Fig. 4g). In summary, the localization is consistent with a role of BdAUX1 in promoting auxin transport from the shoot to the root tip, and in evacuating auxin from the tip via the epidermis. Notably, despite the increased auxin level in *Bdaux1* root tips (Fig. 3a), the *Bdaux1* root agravitropism could be somewhat rescued by application of 1‐NAA (Fig. 5a), similar to *Ataux1* (Swarup *et al*., 2001). However, 1‐NAA levels that rescued agravitropism did not restore normal root elongation (Fig. 5b), which was always higher in *Bdaux1* than in Bd21‐3, indicating that the roles of *BdAUX1* in cell elongation and gravitropism are physiologically separable.

In summary, our detailed analyses of *Bdaux1* mutants revealed phenotypes that are counterintuitive with respect to the expectations set by the precedent of corresponding Arabidopsis mutants. However, interestingly, an exaggerated root elongation phenotype has also been described for *Osaux1* mutants (Yu *et al*., 2015), although it has not been noticed by others working with the same lines (Zhao *et al*., 2015). Moreover, *Osaux1* mutants also display slightly reduced shoot organ elongation (Zhao *et al*., 2015). Yet, compared to the *Bdaux1* mutants, these phenotypes appear relatively mild, and no flower development or reproductive phenotypes were reported. Likewise, *AUX1* mutants in maize and Setaria also display apparently milder inflorescence and root phenotypes than *BdAUX1* (Huang *et al*., 2017). Possibly, this reflects partial genetic redundancy in rice, maize and Setaria, which contain two more *AUX1* homologs than Brachypodium, including close *OsAUX1, ZmAUX1* and *SvAUX1* homologs (Zhao *et al*., 2012, 2015; Huang *et al*., 2017). Thus, the auxin uptake facilitator network in Brachypodium might be less complex than in other grasses, confirming once more that the regulatory wiring of auxin biosynthesis or transport can vary between species, and thus can trigger distinct physiological and morphological consequences if tampered with (Pacheco‐Villalobos *et al*., 2013; O'Connor *et al*., 2014). In summary, our data suggest that in Brachypodium, BdAUX1 primarily assures correct local auxin accumulation and has a broad role in root and shoot development. This role is apparently broader than the role of *AtAUX1* in Arabidopsis, and could potentially encompass activities of *AtLAX* homologs (Marchant *et al*., 2002). However, a detailed analysis of the other Brachypodium AUX1 homologs will be required to conclusively resolve whether this is indeed the case.

## Author contributions

A.v.d.S. and C.S.H. designed the study and wrote the paper. A.v.d.S., C.V., K.L., M.P. and C.S.H. designed experiments. A.v.d.S. and C.V. performed experiments. J.B. and J.V. provided crucial reagents.

## Supporting information

Please note: Wiley Blackwell are not responsible for the content or functionality of any Supporting Information supplied by the authors. Any queries (other than missing material) should be directed to the *New Phytologist* Central Office.

**Fig. S1** Clustal protein sequence alignment of Arabidopsis and Brachypodium AUX1 homologs.
**Fig. S2** Expression analysis of Brachypodium *BdAUX1* and other *AUX1* homologs.
**Fig. S3** Various genetic and physiological analyses of Brachypodium *BdAUX1*.

**Table S1** List of differentially expressed genes in *Bdaux1* root segments (*P *<* *0.01)

**Table S2** Comparison of RNAseq analyses of *Bdaux1* and Bd21‐3 root segments

**Methods S1** DNA sequences of oligonucleotides and the CRISPR/Cas9 cassette used in this study.
