# Supplementary material for: Broad spectrum developmental role of Brachypodium AUX1
Source: New Phytol. 2018 Jun 27;219(4):1216–23. doi: 10.1111/nph.15332 (PMC6100110; doi:10.1111/nph.15332)
Supplement: Supplementary file 1 — Fig. S1 Clustal protein sequence alignment of Arabidopsis and Brachypodium AUX1 homologs. Fig. S2 Expression analysis of Brachypodium BdAUX1 and other AUX1 homologs. Fig. S3 Various genetic and physiological analyses of Brachypodium BdAUX1. [file NPH-219-1216-s001.pdf]

New Phytologist

***New Phytologist* Supporting Information**

Article title: **Broad spectrum developmental role of Brachypodium AUX1**
Authors: Alja van der Schuren, Catalin Voiniciuc, Jennifer Bragg, Karin Ljung, John Vogel, Markus Pauly & Christian S. Hardtke
Article acceptance date: 10 June 2018

The following Supporting Information is available for this article:

Fig. S1 Clustal protein sequence alignment of Arabidopsis and Brachypodium AUX1 homologs.

Fig. S2 Expression analysis of Brachypodium *BdAUX1* and other *AUX1* homologs.

Fig. S3 Various genetic and physiological analyses of Brachypodium *BdAUX1*.

Fig. S1 Clustal protein sequence alignment of Arabidopsis and Brachypodium AUX1 homologs. MView result of multiple sequence alignment. Sequence identities were calculated based on reference sequence AtAUX1 and normalized by aligned length. Amino acids were colored by identity.

Fig. S2 Expression analysis of Brachypodium *BdAUX1* and other *AUX1* homologs. (A) RT-PCR amplification of *BdAUX1* mRNA from wild type and *Bdaux1* mutant background. Different parts of the *BdAUX1* mRNA were amplified using the indicated oligonucleotide pairs. The ca. 600 bp band amplified with oligonucleotides F1 and R1 (middle) corresponded to an apparently unrelated cross-hybridizing artefact (*Bd1g74510*) as determined by Sanger sequencing. The full length cDNA could not be amplified from *Bdaux1* (right) and only produced a small fragment containing 306 bp of exon 7 (as determined by Sanger sequencing), indicating rearrangement of the *BdAUX1* locus by the T-DNA insertion. **(B)** qPCR of the two *BdAUX1* homologs in Bd21-3 and *Bdaux1* roots, relative expression normalized to housekeeping gene *BdUBC18* (error bars = SE). Statistically significant difference compared to wild type is indicated (Student's T-test, a = $p < 0.01$).

Fig. S3 Various genetic and physiological analyses of Brachypodium *BdAUX1*. (A) Primary root length of two Arabidopsis *aux1* mutants as compared to their wild type background Col-0. **(B-C)** Shoot phenotype of indicated complemented and mutant lines. **(D)** Inflorescence phenotype of a homozygous *Bdaux1* mutant created through CRISPR/Cas9 genome editing. **(E)** Monosaccharide analysis of wall material of 1 cm root elongation zone segments (2-day-old roots) of indicated genotypes (error bars = SE). **(F)** Shoot phenotype of indicated genotypes. **(G)** Root length of indicated genotypes. **(H)** Quantification of total cell file number in cross sections of mature tissue layers of indicated genotypes (2-day-old roots). **(I-J)** Root meristems of indicated genotypes (confocal microscopy after ClearSee and calcofluor staining), including parental background controls (I), 2-day-old roots. Statistically significant differences are indicated: (A, E): Student's T-test, a = $p < 0.01$; b = $p < 0.02$; (G-H): ANOVA ($p < 0.001$) & Tukey test, alpha = 0.01. (I-J): composite images.

Figure S1

```
Reference sequence (1): AtAUX1—AT2G38120 Identities normalised by aligned length. Colored by: identity

                         cov    pid   1 [         .         .         .         .         :         .         .         . 80
1 AtAUX1—AT2G38120      100.0% 100.0%   ---MSEGVEAIVANDNGTDQVNGNR----TGKD--NEEHDGSTGSNLSNFLWHGGSVWDAWFSCASNQVAQVLLTLPYSF
2 Bradi2g55340(BdAUX1)   99.4%  81.2%   MVPREHGDEAIVADGNGKEEEVGVMGVGAADGD--EEQHGAGGKFSVTSFLWHGGSVWDAWFSCASNQVAQVLLTLPYSF
3 AtLAX1-AT5G01240       99.0%  81.1%   MSGEKQAEESIVVS--GEDEVAGRKVEDSAAEE--DIDGNGGNGFSMKSFLWHGGSAWDAWFSCASNQVAQVLLTLPYSF
4 Bradi3g21090           96.7%  66.7%   MA----------S-----ETAAGSALADE--KAEAMEQQEAGGKSRLSGLLWHGGSAYDAWFSCASNQVAQVLLTLPYSF
5 AtLAX2-AT2G21050       96.1%  72.7%   -------ME--NGEKAAETVVVGNYVEMEKDGKA------LDIKSKLSDMFWHGGSAYDAWFSCASNQVAQVLLTLPYSF
6 Bradi1g68350           97.7%  67.7%   MAAAANGSL--ADEKAPETIGVGRYVEMEQD-----GNSGSTAKSRLSGLLWHGGSAYDAWFSCASNQVAQVLLTLPYSF
7 AtLAX3-AT1G77690       95.7%  76.6%   -----------MAAEKIETVVAGNYLEMEREEENISGNKKSSTKTKLSNFFWHGGSVYDAWFSCASNQVAQVLLTLPYSF
consensus/100%                          ............s.....p..sG.h................s.t.phpshhWHGGSsaDAWFSCASNQVAQVLLTLPYSF
consensus/90%                           ............s.....p..sG.h................s.t.phpshhWHGGSsaDAWFSCASNQVAQVLLTLPYSF
consensus/80%                           ............st...pp.ssGphh..ttttt....pttusst.ploshhWHGGSsaDAWFSCASNQVAQVLLTLPYSF
consensus/70%                           ...........supttscs.ssGshlt.ptstc...tpptuusKopLSshLWHGGSsaDAWFSCASNQVAQVLLTLPYSF

                         cov    pid  81         .         1         .         .         .         .         :         . 160
1 AtAUX1—AT2G38120      100.0% 100.0%   SQLGMLSGIVLQIFYGLLGSWTAYLISVLYVEYRARKEK-EGKSFKNHVIQWFEVLDGLLGSYWKALGLAFNCTFLLFGS
2 Bradi2g55340(BdAUX1)   99.4%  81.2%   SQLGMLSGVLLQLFYGFLGSWTAYLISVLYVEYRSRKEK-EGVSFKNHVIQWFEVLDGLLGPYWKAAGLAFNCTFLLFGT
3 AtLAX1-AT5G01240       99.0%  81.1%   SQLGMLSGILLQIFYGLMGSWTAYLISVLYVEYRARMEKQEAKSFKNHVIQWFEVLDGLLGPYWKAAGLAFNCTFLLFGS
4 Bradi3g21090           96.7%  66.7%   AQLGMLSGILFQLFYGLLGSWTAYLISILYLEYRTRKEK-DKVDFRNHVIQWFEVLDGLLGRHWRNVGLAFNCTFLLFGS
5 AtLAX2-AT2G21050       96.1%  72.7%   SQLGMLSGILFQLFYGILGSWTAYLISILYVEYRTRKER-EKVNFRNHVIQWFEVLDGLLGKHWRNVGLAFNCTFLLFGS
6 Bradi1g68350           97.7%  67.7%   SQLGMLSGILFQLFYGLMGSWTAYLISILYVEYRTRKER-EKADFRNHVIQWFEVLDGLLGRHWRNVGLAFNCTFLLFGS
7 AtLAX3-AT1G77690       95.7%  76.6%   SQLGMMSGILFQLFYGLMGSWTAYLISVLYVEYRTRKER-EKFDFRNHVIQWFEVLDGLLGKHWRNLGLIFNCTFLLFGS
consensus/100%                          uQLGMhSGllhQlFYGhhGSWTAYLISlLYlEYRsRhE+.-thsF+NHVIQWFEVLDGLLG.aW+shGLhFNCTFLLFGo
consensus/90%                           uQLGMhSGllhQlFYGhhGSWTAYLISlLYlEYRsRhE+.-thsF+NHVIQWFEVLDGLLG.aW+shGLhFNCTFLLFGo
consensus/80%                           SQLGMLSGILhQlFYGlhGSWTAYLISlLYVEYRsRKE+.EthsF+NHVIQWFEVLDGLLG.aW+shGLAFNCTFLLFGS
consensus/70%                           SQLGMLSGILhQLFYGLhGSWTAYLISlLYVEYRoRKE+.EthsF+NHVIQWFEVLDGLLGpaW+slGLAFNCTFLLFGS

                         cov    pid 161         .         .         .         2         .         .         .         . 240
1 AtAUX1—AT2G38120      100.0% 100.0%   VIQLIACASNIYYINDHLDKRTWTYIFGACCATTVFIPSFHNYRIWSFLGLGMTTYTAWYLAIASIIHGQAEGVKHSGPT
2 Bradi2g55340(BdAUX1)   99.4%  81.2%   VIQLIACASNIYYINDRLDKRTWTYIFGACCATTVFIPSFHNYRIWSFLGLGMTTYTAWYLAIAALINGQVEGVTHTGPN
3 AtLAX1-AT5G01240       99.0%  81.1%   VIQLIACASNIYYINDRLDKRTWTYIFGACCATTVFIPSFHNYRIWSFLGLGMTTYTAWYLTIASFLHGQAEGVTHSGPT
4 Bradi3g21090           96.7%  66.7%   VIQLIGCASNIYYVNDHLDKRTWTYIFGACCATTVFIPSFHNYRVWSFLGLLMTTYTAWYIAVASLVHGQVEGVRHSGPT
5 AtLAX2-AT2G21050       96.1%  72.7%   VIQLIACASNIYYINDNLDKRTWTYIFGACCATTVFIPSFHNYRIWSFLGLLMTTYTAWYLTIASILHGQVEGVKHSGPS
6 Bradi1g68350           97.7%  67.7%   VIQLIACASNIYYINDRLDKRTWTYIFGACCATTVFIPSFHNYRIWSFLGLVMTTYTAWYLAIASILHGQVDGVKHSGPT
7 AtLAX3-AT1G77690       95.7%  76.6%   VIQLIACASNIYYINDKLDKRTWTYIFGACCATTVFIPSFHNYRIWSFLGLAMTTYTSWYLTIASLLHGQAEDVKHSGPT
consensus/100%                          VIQLIuCASNIYYlNDpLDKRTWTYIFGACCATTVFIPSFHNYRlWSFLGLhMTTYTuWYlslAuhlpGQs-sVphoGPs
consensus/90%                           VIQLIuCASNIYYlNDpLDKRTWTYIFGACCATTVFIPSFHNYRlWSFLGLhMTTYTuWYlslAuhlpGQs-sVphoGPs
consensus/80%                           VIQLIACASNIYYIND+LDKRTWTYIFGACCATTVFIPSFHNYRIWSFLGLhMTTYTAWYLsIASllHGQsEGVpHSGPo
consensus/70%                           VIQLIACASNIYYIND+LDKRTWTYIFGACCATTVFIPSFHNYRIWSFLGLsMTTYTAWYLsIASllHGQsEGV+HSGPT

                         cov    pid 241         :         .         .         .         .         3         .         . 320
1 AtAUX1—AT2G38120      100.0% 100.0%   KLVLYFTGATNILYTFGGHAVTVEIMHAMWKPQKFKYIYLMATLYVFTLTIPSAAAVYWAFGDALLDHSNAFSLMPKNAW
2 Bradi2g55340(BdAUX1)   99.4%  81.2%   KLVLYFTGATNILYTFGGHAVTVEIMHAMWKPAKFKYIYLLATLYVFTLTLPSASAMYWAYGDELLSHANAFSLLPKTAW
3 AtLAX1-AT5G01240       99.0%  81.1%   KLVLYFTGATNILYTFGGHAVTVEIMHAMWKPRKFKSIYLMATLYVFTLTLPSASAVYWAFGDQLLNHSNAFSLLPKTRF
4 Bradi3g21090           96.7%  66.7%   TIMLYFTGATNILYTFGGHAVTVEIMHAMWRPQKFKAIYLLATLYVLTLTLPSASAAYWAFGDQLLTHSNALSLLPRDAW
5 AtLAX2-AT2G21050       96.1%  72.7%   KLVLYFTGATNILYTFGGHAVTVEIMHAMWKPQKFKSIYLFATLYVLTLTLPSASAVYWAFGDLLLNHSNAFALLPKNLY
6 Bradi1g68350           97.7%  67.7%   KMVLYFTGATNILYTFGGHAVTVEVMHAMWRPQKFKAIYLMATLYVLTLTLPSAASVYWAFGDDLLTHSNALSLLPRTAF
7 AtLAX3-AT1G77690       95.7%  76.6%   TMVLYFTGATNILYTFGGHAVTVEIMHAMWKPQKFKAIYLLATIYVLTLTLPSASAVYWAFGDKLLTHSNALSLLPKTGF
consensus/100%                          phhLYFTGATNILYTFGGHAVTVElMHAMW+PtKFK.IYLhATlYVhTLTlPSAuuhYWAaGD.LLsHuNAhuLhP+sha
consensus/90%                           phhLYFTGATNILYTFGGHAVTVElMHAMW+PtKFK.IYLhATlYVhTLTlPSAuuhYWAaGD.LLsHuNAhuLhP+sha
consensus/80%                           phVLYFTGATNILYTFGGHAVTVEIMHAMW+PpKFK.IYLhATLYVhTLTLPSAuAsYWAFGDtLLsHSNAhSLLP+sta
consensus/70%                           KlVLYFTGATNILYTFGGHAVTVEIMHAMWKPQKFKuIYLhATLYVhTLTLPSASAVYWAFGDpLLsHSNAhSLLPKsua

                         cov    pid 321         .         .         :         .         .         .         .         4 400
1 AtAUX1—AT2G38120      100.0% 100.0%   RDAAVILMLIHQFITFGFACTPLYFVWEKVIGMHDTKSICLRALARLPVVIPIWFLAIIFPFFGPINSAVGALLVSFTVY
2 Bradi2g55340(BdAUX1)   99.4%  81.2%   RDAAVVLMLIHQFITFGFACTPLYFVWEKVIGMHDCKSICLRALARLPIVVPIWFLAIIFPFFGPINSAVGALLVSFTVY
3 AtLAX1-AT5G01240       99.0%  81.1%   RDTAVILMLIHQFITFGFACTPLYFVWEKAIGMHHTKSLCLRALVRLPVVVPIWFLAIIFPFFGPINSAVGALLVTFTVY
4 Bradi3g21090           96.7%  66.7%   RDAAVVLMLIHQFITFGFACTPLYFVWEKLIGLHDCKSLCKRAAARLPVVVPIWFLAIIFPFFGPINSAVGSLLVSFTVY
5 AtLAX2-AT2G21050       96.1%  72.7%   RDFAVVLMLIHQFITFGFACTPLYFVWEKLIGMHECRSMCKRAAARLPVVIPIWFLAIIFPFFGPINSTVGSLLVSFTVY
6 Bradi1g68350           97.7%  67.7%   RDAAVVLMLVHQFITFGFACTPLYFVWEKLIGLHDCRSLCKRAAARLPVVVPIWFLAIVFPFFGPINSAVGSLLVSFTVY
7 AtLAX3-AT1G77690       95.7%  76.6%   RDTAVILMLIHQFITFGFASTPLYFVWEKLIGVHETKSMFKRAMARLPVVVPIWFLAIIFPFFGPINSAVGSLLVSFTVY
consensus/100%                          RDhAVlLMLlHQFITFGFAsTPLYFVWEKhIGhHcs+ShhhRAhsRLPlVlPIWFLAIlFPFFGPINSsVGuLLVoFTVY
consensus/90%                           RDhAVlLMLlHQFITFGFAsTPLYFVWEKhIGhHcs+ShhhRAhsRLPlVlPIWFLAIlFPFFGPINSsVGuLLVoFTVY
consensus/80%                           RDsAVlLMLIHQFITFGFACTPLYFVWEKlIGhH-s+ShChRAhARLPVVlPIWFLAIIFPFFGPINSAVGuLLVSFTVY
consensus/70%                           RDsAVlLMLIHQFITFGFACTPLYFVWEKlIGhH-sKSlChRAhARLPVVVPIWFLAIIFPFFGPINSAVGuLLVSFTVY

                         cov    pid 401         .         .         .         .         :         .         .         . 480
1 AtAUX1—AT2G38120      100.0% 100.0%   IIPSLAHMLTYRSASARQNAAEKPPFFMPSWTAMYVLNAFVVVWVLIVGFGFGGWASVTNFVRQVDTFGLFAKCYQCKPA
2 Bradi2g55340(BdAUX1)   99.4%  81.2%   IIPALAHILTYRTASARANAAEKPPFFLPSWTGMFVLNAFIVVWVFVVGFGLGGWASMVNFIRQIDTFGLFAKCYQCPKP
3 AtLAX1-AT5G01240       99.0%  81.1%   IIPALAHMLTYRTASARRNAAEKPPFFIPSWAGVYVINAFIVVWVLVLGFGFGGWASMTNFIRQIDTFGLFAKCYQCKPP
4 Bradi3g21090           96.7%  66.7%   IIPAMAHMVTFRSPQSRENAVERPPRFAGGWTGAYVINSFVVAWVLVVGFGFGGWASITNFVQQVSTFGLFAKCYQCPPR
5 AtLAX2-AT2G21050       96.1%  72.7%   IIPALAHIFTFRSSAARENAVEQPPRFLGRWTGAFTINAFIVVWVTVVGFGFGGWASMINFVHQIDTFGLFTKCYQCPPP
6 Bradi1g68350           97.7%  67.7%   IIPALAHMITYRSAPARENAVEPPPRFVGRWTGTYMINAFVVAWVLVVGFGFGGWASMTNFIRQIDTFGLFTKCYQCPTT
7 AtLAX3-AT1G77690       95.7%  76.6%   IIPALAHMLTFAPAPSRENAVERPPRVVGGWMGTYCINIFVVVWVFVVGFGFGGWASMVNFVRQIDTFGLFTKCYQCPPH
consensus/100%                          IIPuhAHhhTatss.uRtNAsE.PPhhhstWhuhahlN.FlVsWVhllGFGhGGWAShhNFlpQlsTFGLFsKCYQC...
consensus/90%                           IIPuhAHhhTatss.uRtNAsE.PPhhhstWhuhahlN.FlVsWVhllGFGhGGWAShhNFlpQlsTFGLFsKCYQC...
consensus/80%                           IIPALAHhlTaRousuRpNAsEpPPhFhstWsGhaslNuFlVsWVhlVGFGFGGWAShsNFl+QlDTFGLFsKCYQC.s.
consensus/70%                           IIPALAHMlTaRoAsARcNAsE+PPhFlsuWTGsYsINAFlVVWVhVVGFGFGGWASMsNFlRQIDTFGLFsKCYQCPPs

                         cov    pid 481         .         5         .         .         .         . ] 543
1 AtAUX1—AT2G38120      100.0% 100.0%   AAAAHAPV--SALHHRL----------------------------------------------
2 Bradi2g55340(BdAUX1)   99.4%  81.2%   PVMAAAPS--SSHH-------------------------------------------------
3 AtLAX1-AT5G01240       99.0%  81.1%   PAPIAAGA----HHRR-----------------------------------------------
4 Bradi3g21090           96.7%  66.7%   PAASPFLSPPVAFSPSMPPTPFSFNFTGIFAPMSSTPSPAPAPMPFGLGHHHHRHHRHGL---
5 AtLAX2-AT2G21050       96.1%  72.7%   VMVS-----PPP----ISHPHFN--------------------------------HTHGL---
6 Bradi1g68350           97.7%  67.7%   AQPGL--APPLP----SAAPDASWPFPGVLSNFT-MPAPAPSPAHF---FRHPRHHSHGPALK
7 AtLAX3-AT1G77690       95.7%  76.6%   KP-------------------------------------------------------------
consensus/100%                          ...............................................................
consensus/90%                           ...............................................................
consensus/80%                           s..............................................................
consensus/70%                           sssu...s...s...................................................
```

Figure S2

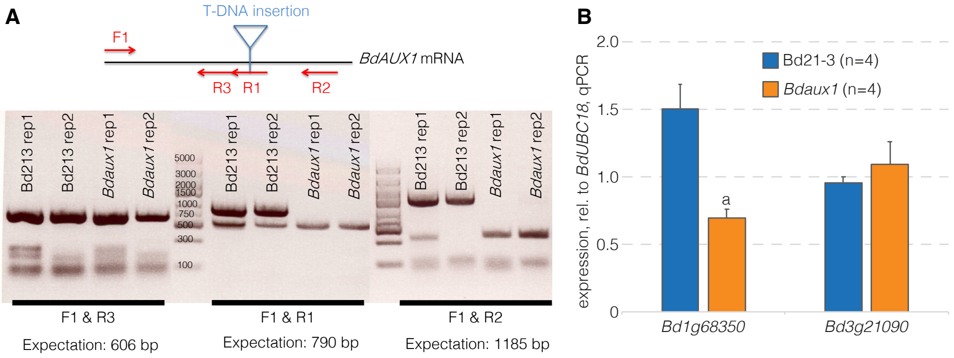
A
T-DNA insertion
F1
BdAUX1 mRNA
R3 R1 R2
Bd213 rep1
Bd213 rep2
Bdaux1 rep1
Bdaux1 rep2
5000
3000
2000
1500
1000
750
500
300
100
F1 & R3
Expectation: 606 bp
F1 & R1
Expectation: 790 bp
F1 & R2
Expectation: 1185 bp
B
expression, rel. to BdUBC18, qPCR
2.0
1.5
1.0
0.5
0
Bd21-3 (n=4)
Bdaux1 (n=4)
a
Bd1g68350
Bd3g21090

Figure S3

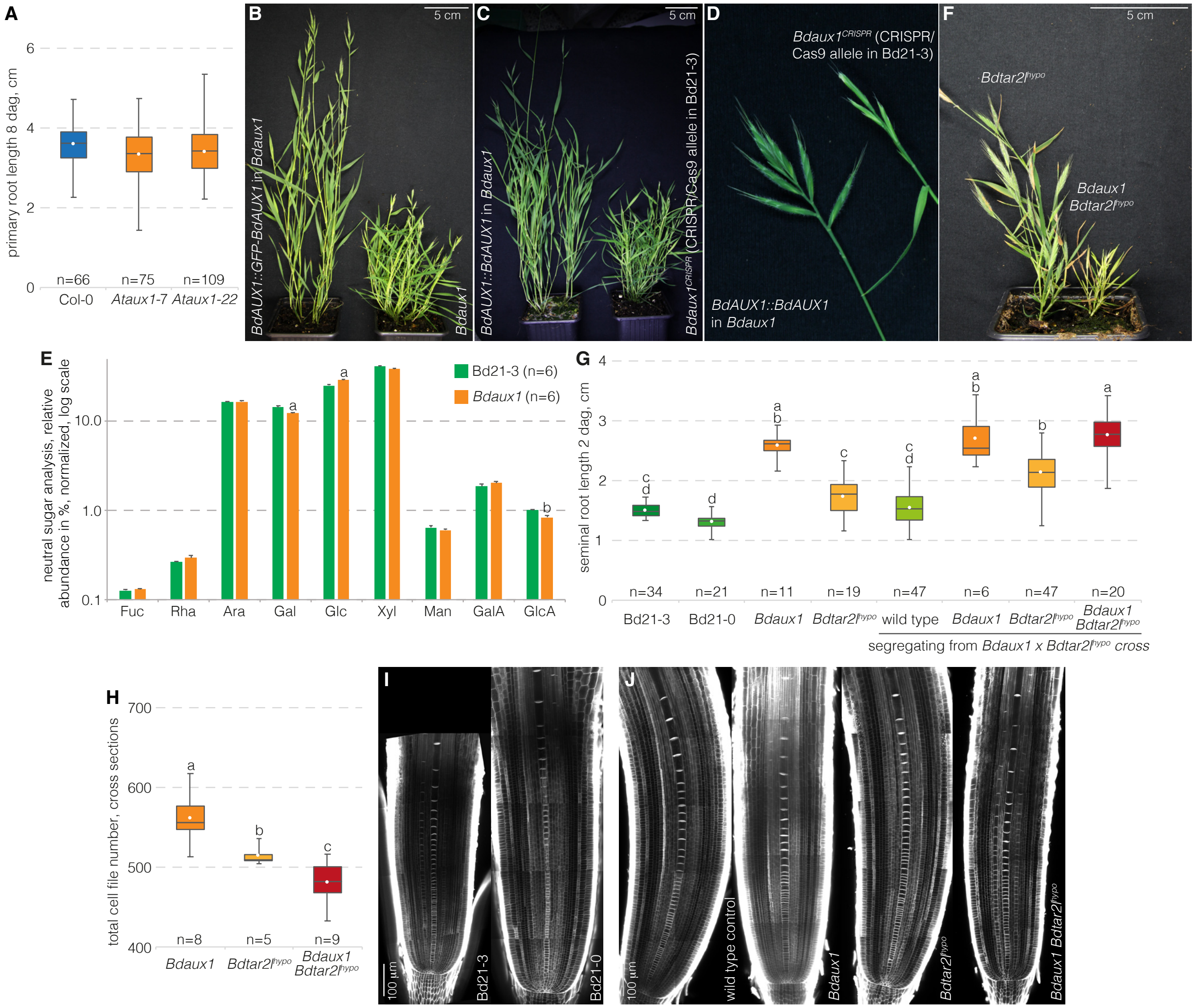

A
primary root length 8 dag, cm
n=66
n=75
n=109
Col-0
Ataux1-7
Ataux1-22
B
5 cm
BdAUX1::GFP-BdAUX1 in Bdaux1
Bdaux1
C
5 cm
BdAUX1::BdAUX1 in Bdaux1
Bdaux1CRISPR (CRISPR/Cas9 allele in Bd21-3)
D
Bdaux1CRISPR (CRISPR/ Cas9 allele in Bd21-3)
BdAUX1::BdAUX1 in Bdaux1
F
5 cm
Bdtar2lhypo
Bdaux1 Bdtar2lhypo
E
neutral sugar analysis, relative abundance in %, normalized, log scale
Bd21-3 (n=6)
Bdaux1 (n=6)
Fuc
Rha
Ara
Gal
Glc
Xyl
Man
GalA
GlcA
G
seminal root length 2 dag, cm
n=34
n=21
n=11
n=19
n=47
n=6
n=47
n=20
Bd21-3
Bd21-0
Bdaux1
Bdtar2lhypo
wild type
Bdaux1
Bdtar2lhypo
Bdaux1 Bdtar2lhypo
segregating from Bdaux1 x Bdtar2lhypo cross
H
total cell file number, cross sections
n=8
n=5
n=9
Bdaux1
Bdtar2lhypo
Bdaux1 Bdtar2lhypo
I
100 µm
Bd21-3
Bd21-0
J
100 µm
wild type control
Bdaux1
Bdtar2lhypo
Bdaux1 Bdtar2lhypo
