# Supplementary material for: Broad spectrum developmental role of Brachypodium AUX1
Source: New Phytol. 2018 Jun 27;219(4):1216–23. doi: 10.1111/nph.15332 (PMC6100110; doi:10.1111/nph.15332)
Supplement: Supplementary file 4 — Methods S1 DNA sequences of oligonucleotides and the CRISPR/Cas9 cassette used in this study. [file NPH-219-1216-s004.pdf]

New Phytologist **Supporting Information**

Article title: **Broad spectrum developmental role of Brachypodium AUX1**

Authors: Alja van der Schuren, Catalin Voiniciuc, Jennifer Bragg, Karin Ljung, John Vogel, Markus Pauly & Christian S. Hardtke

Article acceptance date: 10 June 2018

Supporting Information Methods S1 - DNA and oligonucleotide sequences

Cas9 cassette: FLAG-tag NLS Bd-optimized Cas9

TATGGTACCATGATGATCGACTACAAGGACGACGACGACAAGATGGCCCCGAAGAAGAAGCGCAAGGTGGGCATGGACAAGAAGTA
CTCCATCGGCCTCGACATCGGCACGAACTCCGTGGGCTGGGCCGTGATCACGGACGAGTACAAGGTGCCGTCCAAGAAGTTCAAGG
TGCTCGGCAACACGGACCGCCACTCCATCAAGAAGAACCTCATCGGCGCCCTCCTCTTCGACTCCGGCGAGACGGCCGAGGCCACG
CGCCTCAAGCGCACGGCCCGCCGCCGCTACACGCGCCGCAAGAACCGCATCTGCTACCTCCAGGAGATCTTCTCCAACGAGATGGC
CAAGGTGGACGACTCCTTCTTCCACCGCCTCGAGGAGTCCTTCCTCGTGGAGGAGGACAAGAAGCACGAGCGCCACCCGATCTTCG
GCAACATCGTGGACGAGGTGGCCTACCACGAGAAGTACCCGACGATCTACCACCTCCGCAAGAAGCTCGTGGACTCCACGGACAAG
GCCGACCTCCGCCTCATCTACCTCGCCCTCGCCCACATGATCAAGTTCCGCGGCCACTTCCTCATCGAGGGCGACCTCAACCCGGA
CAACTCCGACGTGGACAAGCTCTTCATCCAGCTCGTGCAGACGTACAACCAGCTCTTCGAGGAGAACCCGATCAACGCCTCCGGCG
TGGACGCCAAGGCCATCCTCTCCGCCCGCCTCTCCAAGTCCCGCCGCCTCGAGAACCTCATCGCCCAGCTCCCGGGCGAGAAGAAG
AACGGCCTCTTCGGCAACCTCATCGCCCTCTCCCTCGGCCTCACGCCGAACTTCAAGTCCAACTTCGACCTCGCCGAGGACGCCAA
GCTCCAGCTCTCCAAGGACACGTACGACGACGACCTCGACAACCTCCTCGCCCAGATCGGCGACCAGTACGCCGACCTCTTCCTCG
CCGCCAAGAACCTCTCCGACGCCATCCTCCTCTCCGACATCCTCCGCGTGAACACGGAGATCACGAAGGCCCCGCTCTCCGCCTCC
ATGATCAAGCGCTACGACGAGCACCACCAGGACCTCACGCTCCTCAAGGCCCTCGTGCGCCAGCAGCTCCCGGAGAAGTACAAGGA
GATCTTCTTCGACCAGTCCAAGAACGGCTACGCCGGCTACATCGACGGCGGCGCCTCCCAGGAGGAGTTCTACAAGTTCATCAAGC
CGATCCTCGAGAAGATGGACGGCACGGAGGAGCTCCTCGTGAAGCTCAACCGCGAGGACCTCCTCCGCAAGCAGCGCACGTTCGAC
AACGGCTCCATCCCGCACCAGATCCACCTCGGCGAGCTCCACGCCATCCTCCGCCGCCAGGAGGACTTCTACCCGTTCCTCAAGGA
CAACCGCGAGAAGATCGAGAAGATCCTCACGTTCCGCATCCCGTACTACGTGGGCCCGCTCGCCCGCGGCAACTCCCGCTTCGCCT
GGATGACGCGCAAGTCCGAGGAGACGATCACGCCGTGGAACTTCGAGGAGGTGGTGGACAAGGGCGCCTCCGCCCAGTCCTTCATC
GAGCGCATGACGAACTTCGACAAGAACCTCCCGAACGAGAAGGTGCTCCCGAAGCACTCCCTCCTCTACGAGTACTTCACGGTGTA
CAACGAGCTCACGAAGGTGAAGTACGTGACGGAGGGCATGCGCAAGCCGGCCTTCCTCTCCGGCGAGCAGAAGAAGGCCATCGTGG
ACCTCCTCTTCAAGACGAACCGCAAGGTGACGGTGAAGCAGCTCAAGGAGGACTACTTCAAGAAGATCGAGTGCTTCGACTCCGTG
GAGATCTCCGGCGTGGAGGACCGCTTCAACGCCTCCCTCGGCACGTACCACGACCTCCTCAAGATCATCAAGGACAAGGACTTCCT
CGACAACGAGGAGAACGAGGACATCCTCGAGGACATCGTGCTCACGCTCACGCTCTTCGAGGACCGCGAGATGATCGAGGAGCGCC
TCAAGACGTACGCCCACCTCTTCGACGACAAGGTGATGAAGCAGCTCAAGCGCCGCCGCTACACGGGCTGGGGCCGCCTCTCCCGC
AAGCTCATCAACGGCATCCGCGACAAGCAGTCCGGCAAGACGATCCTCGACTTCCTCAAGTCCGACGGCTTCGCCAACCGCAACTT
CATGCAGCTCATCCACGACGACTCCCTCACGTTCAAGGAGGACATCCAGAAGGCCCAGGTGTCCGGCCAGGGCGACTCCCTCCACG
AGCACATCGCCAACCTCGCCGGCTCCCCGGCCATCAAGAAGGGCATCCTCCAGACGGTGAAGGTGGTGGACGAGCTCGTGAAGGTG
ATGGGCCGCCACAAGCCGGAGAACATCGTGATCGAGATGGCCCGCGAGAACCAGACGACGCAGAAGGGCCAGAAGAACTCCCGCGA
GCGCATGAAGCGCATCGAGGAGGGCATCAAGGAGCTCGGCTCCCAGATCCTCAAGGAGCACCCGGTGGAGAAACGCAGCTCCAGAA
CGAGAAGCTCTACCTCTACTACCTCCAGAACGGCCGCGACATGTACGTGGACCAGGAGCTCGACATCAACCGCCTCTCCGACTACG
ACGTGGACCACATCGTGCCGCAGTCCTTCCTCAAGGACGACTCCATCGACAACAAGGTGCTCACGCGCTCCGACAAGAACCGCGGC
AAGTCCGACAACGTGCCGTCCGGGAGGTGGTGAAGAAGATGAAGAACTACTGGCGCCAGCTCCTCAACGCCAAGCTCATCACGCAG
CGCAAGTTCGACAACCTCACGAAGGCCGAGCGCGGCGGCCTCTCCGAGCTCGACAAGGCCGGCTTCATCAAGCGCCAGCTCGTGGA
GACGCGCCAGATCACGAAGCACGTGGCCCAGATCCTCGACTCCCGCATGAACACGAAGTACGACGAGAACGACAAGCTCATCCGCG

AGGTGAAGGTGATCACGCTCAAGTCCAAGCTCGTGTCCGACTTCCGCAAGGACTTCCAGTTCTACAAGGTGCGCGAGATCAACAAC
TACCACCACGCCCACGACGCCTACCTCAACGCCGTGGTGGGCACGGCCCTCATCAAGAAGTACCCGAAGCTCGAGTCCGAGTTCGT
GTACGGCGACTACAAGGTGTACGACGTGCGCAAGATGATCGCCAAGTCCGAGCAGGAGATCGGCAAGGCCACGGCCAAGTACTTCT
TCTACTCCAACATCATGAACTTCTTCAAGACGGAGATCACGCTCGCCAACGGCGAGATCCGCAAGCGCCCGCTCATCGAGACGAAC
GGCGAGACGGGCGAGATCGTGTGGGACAAGGGCCGCGACTTCGCCACGGTGCGCAAGGTGCTCTCCATGCCGCAGGTGAACATCGT
GAAGAAGACGGAGGTGCAGACGGGCGGCTTCTCCAAGGAGTCCATCCTCCCGAAGCGCAACTCCGACAAGCTCATCGCCCGCAAGA
AGGACTGGGACCCGAAGAAGTACGGCGGCTTCGACTCCCCGACGGTGGCCTACTCCGTGCTCGTGGTGGCCAAGGTGGAGAAGGGC
AAGTCCAAGAAGCTCAAGTCCGTGAAGGAGCTCCTCGGCATCACGATCATGGAGCGCTCCTCCTTCGAGAAGAACCCGATCGACTT
CCTCGAGGCCAAGGGCTACAAGGAGGTGAAGAAGGACCTCATCATCAAGCTCCCGAAGTACTCCCTCTTCGAGCTCGAGAACGGCC
GCAAGCGCATGCTCGCCTCCGCCGGCGAGCTCCAGAAGGGCAACGAGCTCGCCCTCCCGTCCAAGTACGTGAACTTCCTCTACCTC
GCCTCCCACTACGAGAAGCTCAAGGGCTCCCCGGAGGACAACGAGCAGAAGCAGCTCTTCGTGGAGCAGCACAAGCACTACCTCGA
CGAGATCATCGAGCAGATCTCCGAGTTCTCCAAGCGCGTGATCCTCGCCGACGCCAACCTCGACAAGGTGCTCTCCGCCTACAACA
AGCACCGCGACAAGCCGATCCGCGAGCAGGCCGAGAACATCATCCACCTCTTCACGCTCACGAACCTCGGCGCCCCGGCCGCCTTC
AAGTACTTCGACACGACGATCGACCGCAAGCGCTACACGTCCACGAAGGAGGTGCTCGACGCCACGCTCATCCACCAGTCCATCAC
GGGCCTCTACGAGACGCGCATCGACCTCTCCCAGCTCGGCGGCGACTAGCTGCTTTAATGAGATATGCGAGACGCCTATGATCGCA
TGATATTTGCTTTCAATTCTGTTGTGCACGTTGTAAAAACCTGAGCATGTGTAGCTCAGATCCTTACCGCCGGTTTCGGTTCATTC
TAATGAATATATCACCCGTTACTATCGTATTTTTATGAATAATATTCTCCGTTCAATTTACTGATTGTACCCTACTACTTATATGT
ACAATATTAAAATGAAAACAATATATTGTGCTGAATAGGTTTATAGCGACATCTATGATAGAGCGCCACAATAACAAACAATTGCG
TTTTATTATTACAAATCCAATTTTCGGGGATCCTCTAGAGTCGACCTGCAGGCATGCAAGCTTGGCACGGTTACCAAGCGCGCAAA

***BdAUX1* k.o. sgRNA cassette: *BdU6* promoter tracrRNA *OsU6* promoter BsaI or BtgZI sites**

AAAGGATCCGGCGCGCCTACTTGGGCTGTTGCTCTCTACTGGGTTGGGCCGCATGGACTGACACAGGCCCACGCGCGGTCCTTCAC
GAGCCTGGGGCTGGCCTGATCCGATGGTTGCTGATCAAGGCAACAGGCTAGAAAGTTTAGTCCCACCTCGCGAGATGAAGGATAGT
TTGACTAGATTATAAACATTCTGCTACCACCCTTCTCagagaccgagctcggtctcaGTTTTAGAGCTAGAAATAGCAAGTTAAAA
TAAGGCTAGTCCGTTATCAACTTGAAAAAGTGGCACCGAGTCGGTGCTTTTTTTGAGATTTCCAACCAGGTCCCTGGAGCCCATA
GTCTAGTAACGGCCGCCAGTGTGCTGGAATTGCCCTTGGATCATGAACCAACGGCCTGGCTGTATTTGGTGGTTGTGTAGGGAGAT
GGGGAGAAGAAAAGCCCGATTCTCTTCCTGTGATGGGCTGGATGCATGCGGGGGAGCGGGAGGCCCAAGTACGTGCACGGTGAGCG
GCCCACAGGGCGAGTGTGAGCGCGAGAGGCGGGAGGAACAGTTTAGTACCACATTGCCCAGCTAACTCGAACGCGACCAACTTATA
AACCCGCGCGCTGTCGCTTGTGTacaattgcatcatcgcagtcagcgatgagtacagcaaGTTTTAGAGCTAGAAATAGCAAGTTA
AAATAAGGCTAGTCCGTTATCAACTTGAAAAAGTGGCACCGAGTCGGTGCTTTTTTTGAGATTTTGCGCACCGGTCCTGCAGGGTC
AAGCTTAAA

List of primers:

pUbi1-For	5'-GAG CTC CAG CTT GCA TGC CTG CAG TG-3'
pUbi1-Rev	5'-GAG CTC TCT AGA GTC GAC CTG CAG AA-3'
sgRNA-BdAux1a F2	5'-TCT CGT CAC CAG CTT CCT CTG GCA-3'
sgRNA-BdAux1a R	5'-AAA CTG CCA GAG GAA GCT GGT GAC-3'
sgRNA-BdAux1b F	5'-GTG TGA TCC GGT AGT TGT GGA AGG-3'
sgRNA-BdAux1b R	5'-AAA CCC TTC CAC AAC TAC CGG ATC-3'
BdAUX1L-WT-F	5'-GTG AAC TTT CCA CAC TGA GC-3'
BdAUX1L-WT-R	5'-TCA CAA GAG CTG GGC AAT GG-3'
pJJ2LBA-RB-F2	5'-CAG GAA TTC ATG CCG ACA GC-3'
KanR-XhoI-For	5'-CCA CTC GAG GAT CTC CAC TCT AGT CGA G-3'
KanR-XhoI-Rev	5'-TGT CTC GAG TTG AAC GAT CGG GGA TCC-3'
promBdAUX1F2SacI	5'-CTA GAG CTC TGG ACG TGG TTT TGT CCT AG-3'
promBdAUX1R2SalI	5'-ACG CGT CGA CAT CTC TTC AAC GCG CTG TC-3'
pCAMBIA-promBdAUX1F	5'-CAT GAT TAC GAA TTC GAG CTC GTC ACT TAA TCT CGT C-3'
promBdAUX1seq6R	5'-CGA ATT TCC TCT CTG TCT CC-3'
promBdAUX1seq6F	5'-GGA GAC AGA GAG GAA ATT CG-3'

genBdAUX1seq5R	5'-TGG AAC GAT GAG GTG CAT TG-3'
genBdAUX1seq5F	5'-CAA TGC ACC TCA TCG TTC CA-3'
genBdAUX1R3-pCAMBIA	5'-GGA AAT TCG AGC TGG TCA CCT AGC AAG CAT TAC TGG GTT-3'
BdAUX1p-XmaI F	5'-GCG ACT GTG CCA ACA CCC-3'
BdAUX1p-sGFP R	5'-GCC CTT GCT CAC CAT CTC TTC AAC GCG CTG TCC TC-3'
sGFP(S65T) F	5'-ATG GTG AGC AAG GGC GAG G-3'
sGFP+linker R	5'-ATC CTC TAG AGT CGA CCT TGT ACA GCT CGT CCA TGC-3'
sGFPlink-AUX1g F	5'-GTC GAC TCT AGA GGA TCC ATG GTG CCG CGC GAG CAT-3'
AUX1g-PacI-pCAM R	5'-TTT TTC CTC GGG TTA GTT AAT TAA TTC-3'
